# Breaking the Limit of Micro‐Ductility in Oxide Glasses

**DOI:** 10.1002/advs.201901281

**Published:** 2019-07-24

**Authors:** Kacper Januchta, Malwina Stepniewska, Lars R. Jensen, Yang Zhang, Marcel A. J. Somers, Mathieu Bauchy, Yuanzheng Yue, Morten M. Smedskjaer

**Affiliations:** ^1^ Department of Chemistry and Bioscience Aalborg University Fredrik Bajers Vej 7H 9220 Aalborg East Denmark; ^2^ Department of Materials and Production Aalborg University Fibigerstræde 16 9220 Aalborg East Denmark; ^3^ Department of Mechanical Engineering Technical University of Denmark Produktionstorvet 425 2800 Kongens Lyngby Denmark; ^4^ Department of Civil and Environmental Engineering University of California Los Angeles 7400 Boelter Hall Los Angeles CA 90095 USA

**Keywords:** crack resistance, deformation, glasses, indentation, micro‐ductility

## Abstract

Oxide glasses are one of the most important engineering and functional material families owing to their unique features, such as tailorable physical properties. However, at the same time intrinsic brittleness has been their main drawback, which severely restricts many applications. Despite much progress, a breakthrough in developing ultra‐damage‐resistant and ductile oxide glasses still needs to be made. Here, a critical advancement toward such oxide glasses is presented. In detail, a bulk oxide glass with a record‐high crack resistance is obtained by subjecting a caesium aluminoborate glass to surface aging under humid conditions, enabling it to sustain sharp contact deformations under loads of ≈500 N without forming any strength‐limiting cracks. This ultra‐high crack resistance exceeds that of the annealed oxide glasses by more than one order of magnitude, making this glass micro‐ductile. In addition, a remarkable indentation behavior, i.e., a time‐dependent shrinkage of the indent cavity, is demonstrated. Based on structural analyses, a molecular‐scale deformation model to account for both the ultra‐high crack resistance and the time‐dependent shrinkage in the studied glass is proposed.

## 1 Introduction

Enhancement and understanding of the mechanical properties of glasses, particularly oxide glasses, is a challenging and critical topic in materials research.^1^, ^2^ Brittleness of glass has been the major hindrance for many engineering and functional applications for centuries.^3^, ^4^, ^5^ However, it is challenging to drastically reduce their brittleness despite many efforts, because the crack tip formation and crack growth mechanisms have not been thoroughly understood. To overcome the inherent brittleness of oxide glasses, several post‐processing approaches, such as thermal tempering,^6^ chemical strengthening,^7^, ^8^ or crack‐sealing particle inclusion^9^ have been attempted, with the aim to augment the critical stress for crack initiation and to retard the rate of crack growth by counteracting the stresses acting on the crack tip. Meanwhile, molecular dynamics simulations have suggested that, based on their composition, some selected macroscopically brittle silicate glasses can exhibit some nanoscale ductility.^10^ Recent compressive loading experiments have also shown that sufficiently small (µm size) specimens of silica can undergo extensive strain without fracturing.^11^, ^12^ However, intrinsically ductile, bulk oxide glasses are yet to be developed.

An appealing strategy to overcome the limitations of brittleness is to enhance the damage or crack resistance of glass by tuning the network structure through composition design, i.e., a rational design of material composition with a specific set of properties based on previously acquired knowledge. There are several recent examples in using this strategy, e.g., silicon oxycarbides,^13^ lithium aluminoborates,^14^ and binary aluminosilicates synthesized using container‐less melting technique.^15^ Here, we refer to “crack resistance” as the ability of the material to withstand loads without initiation of cracks upon contact with a sharp object (i.e., indentation).^16^ Such measure of local brittleness is of practical importance, since radial cracks, propagating perpendicular to the glass surface (and induced by contact damage), are the strength‐limiting factor for many applications.^17^ The radial cracks occur because of residual stresses stored around the deformation zone during indentation,^17^, ^18^ so much research has been concentrated on designing compositions that facilitate local densification underneath the indenter as an effective means of stress‐dissipation. The reduction of the residual stress can be achieved by designing glasses with large free volume^13^, ^19^ or with self‐adaptive networks^14^ where structural rearrangements promote densification, by controlling rigidity fluctuations at the nanoscale,^20^ or by shear‐induced plasticity.^21^, ^22^, ^23^ Glasses with strong oxide bonds may also exhibit high resistance to crack initiation upon contact loading,^15^ presumably due to their high fracture surface energy.^24^

In this work, we discover that a caesium aluminoborate glass exhibits superior crack resistance and micro‐ductility when it is subjected to surface aging. In this composition, Al and B serve the roles as glass network formers, while Cs is the network modifier. As we have shown in our previous work,^14^, ^25^, ^26^ aluminoborate glasses generally exhibit high resistance to indentation cracking, including the present Cs_2_O‐Al_2_O_3_‐B_2_O_3_ composition.^25^, ^27^ In a strong contrast to the previous work, we here show that crack‐free Vickers indents at loads as high as 490 N (50 kgf) can be generated by subjecting the caesium glass surface to humid aging, whereas only around 30 N loading was possible in freshly polished samples of the same composition reported elsewhere.^25^ This significant advancement is made possible based on the following four principles for glass composition design. First, the composition should include the network‐forming ions that can coordinate with more oxygen anions under stress, which allows them to dissipate the mechanical work stored during a contact event, and thereby to reduce the crack probability.^14^ To fulfill this criterion, aluminum and boron will be ideal network formers unlike, e.g., silicon.^28^ Second, to further lower the residual stress upon contact loading, the shear‐induced plasticity should also be promoted.^21^, ^23^ The shear deformation can be enhanced by reduction of network connectivity, enabling the studied glasses to suffer less from the stresses that induce cracks.^22^ For this purpose, Cs is an ideal choice due to its highly ionic bonds with oxygen. The low network rigidity of the present is evidenced by its very low hardness value (*H*_V_ = 2.0 GPa), since hardness and network rigidity are directly correlated in oxide glasses.^2^ Third, the composition should have a network modifier/former ratio that limits the number of nonbridging oxygens,^22^ yet preserves good glass‐forming ability. This is achieved by having a slightly peralkaline composition, i.e., higher fraction of Cs than Al.^29^ Fourth, the chemical durability of the glass should be relatively low to facilitate rapid hydration of the glass surface, and thereby significantly increase the crack resistance of the glass as shown in the Results section. Such hydration enables plastic deformation at room temperature of the surface layer, as also evidenced by the recovery of small indent cavities under ambient conditions.^25^ The very high polarizability of the Cs^+^ ion facilitates this behavior. As a result of these principles, the present glass is characterized by a very high Poisson's ratio and very low hardness compared to other known oxide glasses.^30^ Typically, these properties are needed for metallic glasses to exhibit intrinsic ductility.^1^, ^3^ Moreover, the caesium aluminoborate glass can be easily synthesized via melt‐quenching, and displays relaxation of the deformation zone.^25^ Based on a detailed indentation study (including effects of loading up to 50 kgf, indenter tip sharpness, loading rate, etc.) and additional laser microscopy and micro‐Raman spectroscopy experiments, we here propose a molecular mechanism, involving a load‐induced reorganization of the structural units followed by hydration, to account for the indent cavity recovery (self‐healing) and ultra‐high resistance to indentation cracking; two unique features of the present aged glass composition, to the best of our knowledge. This work thus paves the way for the compositional design of damage‐resistant, and potentially ductile, oxide glass compositions.

## 2 Results

### 2.1 Glass Formation and Structure

Melt‐quenching of the investigated composition (25Cs_2_O–20Al_2_O_3_–55B_2_O_3_, in mol%) results in a glass without a long‐range‐ordered structure according to X‐ray diffraction (Figure S1 in the Supporting Information) and a glass transition temperature *T*_g_ of 403 °C (Figure S2 in the Supporting Information). The resulting glass is transparent in the wavelength range of visible light (Figure S3 in the Supporting Information) and compositionally homogenous at the microscale, as evidenced by identical micro‐Raman scattering spectra acquired at different surface locations (Figure S4 in the Supporting Information). The caesium aluminoborate glass features low hardness (*H*_V_ = 2.0 GPa) and low Young's modulus (*E* = 20 GPa) but high Poisson's ratio (ν = 0.32), compared to other oxide glasses.^30^ **Table** **1** shows a comparison of some characteristics between the present glass and two reference materials, viz., a commercial soda‐lime‐silica window glass (13Na_2_O–6MgO–10CaO–71SiO_2_, in mol%) and a model aluminosilicate cover glass (12Na_2_O–6MgO–18Al_2_O_3_–6B_2_O_3_–58SiO_2_, in mol%).^31^ The low hardness and stiffness, as well as the relatively poor chemical durability (Figure S5 in the Supporting Information), of the present glass are a direct consequence of its atomic structure.

**Table 1:** Glass transition temperature (*T*_g_), density (ρ), Vickers hardness measured at 1 N (*H*_V_), Young's modulus (*E*), shear modulus (*G*), and Poisson's ratio (ν) for the present caesium aluminoborate glass and two reference glasses: a soda‐lime‐silica^30^ (13Na_2_O–6MgO–10CaO–71SiO_2_, in mol%) and an aluminosilicate^31^ (12Na_2_O–6MgO–18Al_2_O_3_–6B_2_O_3_–58SiO_2_, in mol%)

	*T*_g_ [°C]	ρ [g cm^−3^]	*H*_v_ [GPa]	*E* [GPa]	*G* [GPa]	ν [–]
Soda‐lime‐silica	562	2.53	5.8	72	29	0.23
Aluminosilicate	671	2.44	6.6	74	30	0.24
Cs‐aluminoborate	403	2.89	2.0	20	8	0.32

The skeleton of the glass network is composed of B and Al atoms linked by O atoms, wherein Cs cations fill in the voids. According to solid‐state NMR spectroscopy experiments,^25^ both trigonal (92%) and tetrahedral (8%) boron species are present in the glass, while the Al atoms reside in predominantly tetrahedral configuration (97%), with the remaining Al in five‐ and sixfold coordination. Although the aluminoborate network consists mostly of rather strong Al‐O and B‐O bonds,^32^ the low connectivity of the B atoms (i.e., most threefold coordination with O atoms) yields a low‐dimensional atomic network, which, in turn, promotes the overall flexibility of the glass. This ensures the ability to increase the coordination number of B and Al cations under stress, which allows the glass to dissipate the mechanical work stored during a contact event, and thereby to reduce the crack probability.^14^ The Cs‐O bonds are highly ionic and exhibit low binding energy,^32^ thereby creating weak points in the structure, which facilitate easy initiation of shear deformation. This shear deformation enables the glass to easily dissipate stress, leading to the ability to withstand higher loads without fracturing.

### 2.2 Resistance to Indentation Cracking

The most striking feature of the present aluminoborate glass is its extremely high resistance to crack initiation. Here, we focus on the resistance to radial crack formation, since lateral cracks that propagate parallel to the surface typically remain inside the glass and thus do not have a pronounced impact on strength.^17^ First, we consider indentation tests using different diamond tips, namely, sphere, semi‐sharp 136° Vickers, and sharp cube corner geometries. To mimic the damage conditions experienced in the field, we perform indentation under constant ambient conditions (Experimental Section). The surface of the caesium aluminoborate glass was polished immediately prior to indentation to allow for direct comparison under different testing conditions, as water is known from previous work to facilitate crack formation in oxide glasses.^33^, ^34^ This is particularly important for the aluminoborate glass, as the chemical durability of the reference silicate and aluminosilicate glasses (used for comparison) are much higher than that of the aluminoborate glass.

**Figure 1:**
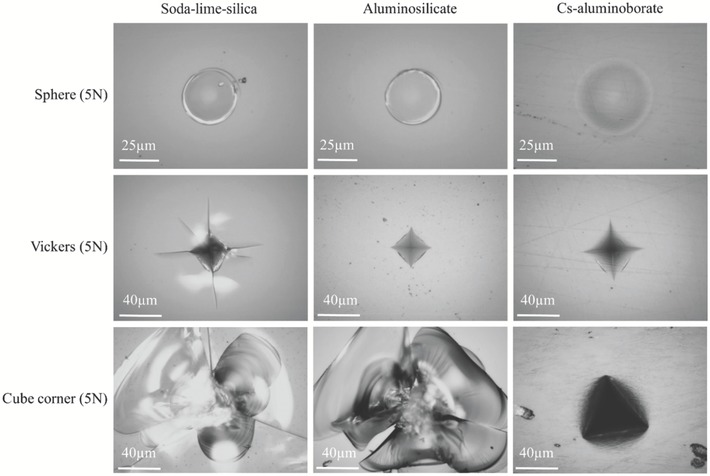
Indentation cracking patterns of different oxide glasses using different indenter tip geometries. From left: soda‐lime‐silica, aluminosilicate glass, and Cs‐aluminoborate glass. From top: sphere, Vickers, and cube corner geometry at 5 N load. Increasing the sharpness of the indenter tip facilitates cracking, and the resistance to crack initiation increases in the order from soda‐lime‐silica to aluminosilicate to Cs‐aluminoborate.

The annealed aluminoborate glass, which is free of any compressive or tensile stresses, is able to withstand much higher loads without radial cracking compared to both soda‐lime‐silica and the more crack‐resistant aluminosilicate glass. **Figure** **1** shows that increasing the sharpness of the indenter tip increases the probability for crack formation, in agreement with a previous study,^35^ as the sharper tip leaves less room for densification and increases the residual stress.^17^ The crack initiation probability at a given load decreases in the order from soda‐lime‐silica through aluminosilicate to aluminoborate glass. Interestingly, cube corner indentation at 5 N does not lead to any crack formation in the present aluminoborate glass. This is an important observation, considering that ≈100 mN is sufficient to induce radial cracking in otherwise highly crack‐resistant calcium aluminoborosilicate glasses.^36^

**Figure 2:**
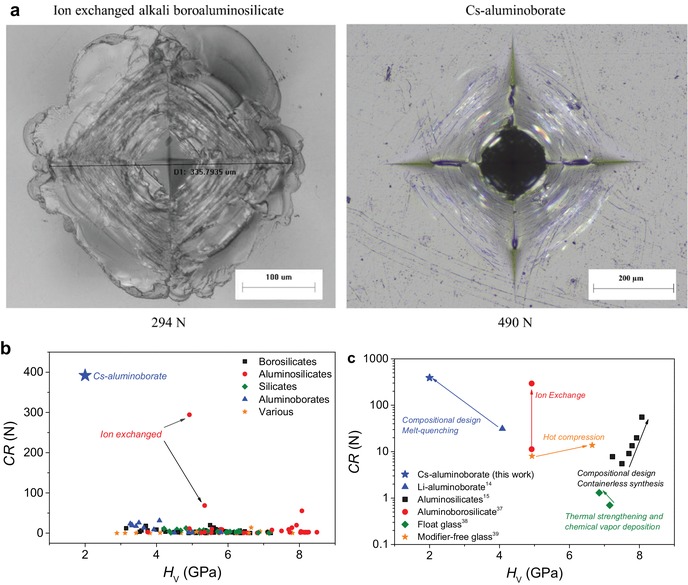
a) Comparison of Vickers indents produced in an ion‐exchanged alkali boroaluminosilicate glass at 294 N (30 kgf) and the present Cs‐aluminoborate glass at 490 N (50 kgf). The former image is reproduced from Gross and Price^37^ under the Creative Commons and Attributions License (CC BY). The cracking patterns are visually similar, with neither of the indents showing any sign of radial cracking. The centers of both indents appear undamaged as their large depths make them difficult to capture by optical microscopy. b) Correlation between crack resistance (CR) and Vickers hardness (*H*_V_) for oxide glasses. The CR of the present glass is estimated to be an order of magnitude higher than those of previously reported annealed glasses (references listed in the Supporting Information). Data for the ion‐exchanged glass from (a) as well as ion‐exchanged soda‐lime‐silica are also shown. c) CR versus *H*_V_ plot showing the effect of various strengthening strategies to design more crack‐resistant glasses, including data for ion exchange,^37^ chemical vapor deposition combined with thermal strengthening,^38^ hot compression,^39^ and compositional design.^14^, ^15^

Next, we consider Vickers indentation experiments at higher loads to probe the crack initiation threshold for the caesium aluminoborate glass. Remarkably, the glass is able to survive Vickers indents at a very high load of 490 N (50 kgf) without forming any radial cracks under ambient conditions (**Figure** **2**a). This is possible when the glass has first been aged, i.e., stored in ambient conditions for 7 days and not polished immediately before indentation. When the glass is polished immediately before indentation, the resistance to cracking is lowered significantly, down to around 30 N, in agreement with our previous reports on this glass composition without surface aging.^25^, ^26^ These observations demonstrate that exposure to humid air has a positive impact on the damage resistance of the present glass, unlike the behavior of other known oxide glasses.^33^, ^34^ The result also infers that the glass exhibits a ductile behavior at the microscale, since it is able to deform permanently to large strain values (≈0.6 mm indent diagonal) without fracture. To test whether the micro‐ductility of this glass is solely due to its high Poisson's ratio (0.32), which is close to the proposed threshold for a brittle‐to‐ductile transition in metallic glasses,^1^ we have designed and tested another oxide glass (lanthanum zinc borate) with similar high Poisson's ratio (see Supporting Methods in the Supporting Information). This glass, however, displays much lower threshold (≈4 N) for Vickers indentation cracking (Figure S6 in the Supporting Information).

The ultra‐high threshold for indentation cracking in the present glass has thus far only been observed for commercially optimized compositions that are subjected to K^+^‐for‐Na^+^ ion exchange. For comparison, in Figure 2a, we have included a 294 N (30 kgf) Vickers indent of a chemically strengthened aluminoborosilicate glass, which has a 55 µm deep compressive layer at the surface with a peak stress of 715 MPa.^37^ The vast majority of crack resistance data (i.e., load resulting in a 50% probability of radial crack formation) for annealed oxide glasses is below 10 N (Figure 2b). In the case of the caesium aluminoborate, the crack resistance is difficult to quantify accurately, because the probability for crack initiation and the resulting shapes of the indents are highly sensitive to humidity, as well as loading conditions (Figure S7 in the Supporting Information). Given the observation of a crack‐free Vickers indent at 490 N (Figure 2a) and based on our various measurements conditions, we estimate the average crack resistance to be at least around 400 N (Figure 2b). This is a record value for annealed oxide glasses, i.e., glasses that have not undergone any extrinsic strengthening procedure. Independent of the actual crack resistance value, the present observation of crack‐free indents at the very high loads shows that rational design of the chemical composition is a powerful strategy to improve the damage resistance, even compared with other methods such as ion exchange, thermal strengthening, or hot compression (Figure 2c).^37^, ^38^, ^39^

### 2.3 Volume Recovery of Indentation Cavities

**Figure 3:**
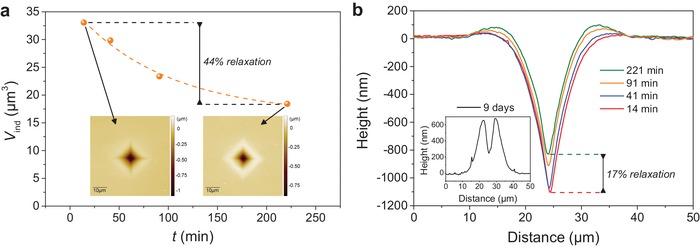
a) Indent cavity volume (*V*_ind_) as a function of time (*t*) after unloading. The Vickers indentation impressions were produced at a 0.98 N load and *V*_ind_ was extracted from the topographical images (insets) acquired using laser microscopy. At least 44% of the initial value of *V*_ind_ is recovered within 221 min after unloading. b) Cross‐section profiles extracted from the images of the indents aged for various durations: 14, 41, 91, and 221 min (main figure), and 9 days (inset). At least 17% of the depth is recovered within 221 min after unloading.

Besides its ultra‐high resistance to crack initiation, the present glass also exhibits an unusual change in shape and size of the indentation impressions as a function of time, as detected by optical microscopy (Figure S8 in the Supporting Information). As the topography of the indent site is continuously changing during imaging, we cannot apply the traditional method proposed by Yoshida et al.^40^ for quantification of the densification contribution to the indentation volume based on annealing and volume recovery experiments. In the present glass, the indent shrinkage (or relaxation) continues even several days after unloading. The rate of the relaxation has been quantified by using laser microscopy to acquire topographical images of Vickers impressions of the glasses that have been aged (room temperature, ≈40% RH) for different durations (**Figure** **3**a). A 0.98 N indent Vickers impression is able to recover 17% of its depth and 44% of its volume within 4 h (Figure 3a,b). Impressions produced at higher indentation loads exhibit smaller relative recovery values (Figure S9 in the Supporting Information).

Nine days after unloading, we have observed a 0.98 N Vickers indent with a topography that significantly differs from those exhibited shortly after unloading, making quantification of the volume recovery difficult (see its cross‐section profile in the inset of Figure 3b). Astonishingly, the entire cavity has recovered above the glass surface, with substantial amount of protrusion surrounding the point of indenter contact. In other words, the material does not merely recover to its original (i.e., unindented) state, but rather continuously expands. This behavior suggests that swelling, presumably linked to atomic‐scale reorganization of the structure, is occurring. The material surrounding the indentation cavity thus undergoes certain structural changes due to a release of the high magnitude of stresses developed during the sharp contact loading. Structural changes of the affected material can lead to changes in mechanical properties, such as hardness and chemical durability.^41^, ^42^ Niu et al. reported a weakening of the silicate network following indentation, as the dissolution rate of the material surrounding the indent cavity was higher than that of a nonloaded surface in both soda‐lime‐silica and pure silica glasses.^42^ By placing a small indent close to the edge of a pre‐existing larger indent, Gross and Tomozawa showed that hardness depends on the fictive temperature of the glass, and that the magnitude of the change in hardness depends on the chemical composition of the glass.^41^ In the case of the present glass, we find that *H*_V_ of the slowly recovered material (i.e., in the near vicinity of a pre‐existing edge) is not different from that of the unaffected material (i.e., where the distance to the pre‐existing indent edge is large). On the other hand, the shapes of these microindents differ from each other (Figure S10 in the Supporting Information), demonstrating that the post‐indentation recovery leads to a change in the deformation mechanism.

### 2.4 Stress‐Assisted Network Hydrolysis

**Figure 4:**
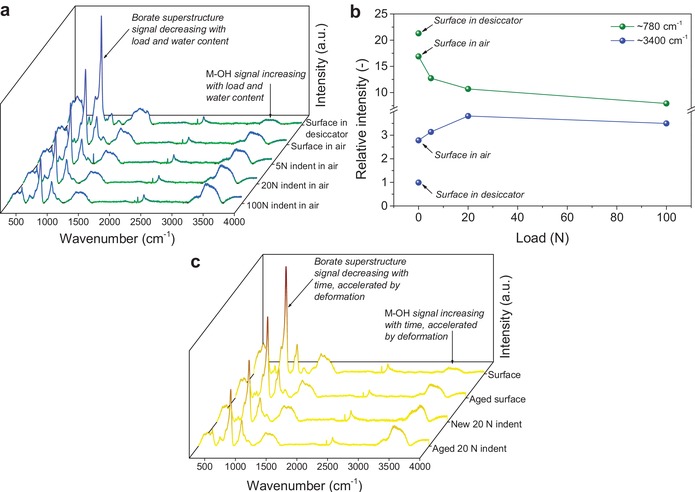
a) Raman spectra acquired on the surface of the glass aged in air and in a desiccator, and in the center of Vickers indents produced at different loads aged in air. The aging time was 7 days in each case at ≈50% RH. The structure of the pristine glass surface changes upon exposure to humid air, and the effect of humidity is enhanced by indentation. b) Effect of indentation load and aging on the intensity of the two Raman bands at ≈780 and ≈3400 cm^−1^ calculated relative to the intensity of the Raman 2330 cm^−1^ band, since the intensity of the latter is not affected by indentation or aging. The broad envelope at ≈3400 cm^−1^ (due to hydrolyzed species) increases in intensity, while the sharp envelope at ≈780 cm^−1^ (due to various borate superstructures) decreases in intensity with increasing load and with water availability. c) Raman spectra acquired on freshly polished surface of the glass, the surface aged in air, in the center of a 20 N indent placed in the aged surface, and in the center of an aged 20 N indent placed in the freshly polished surface. All the spectra were normalized with respect to the total integrated area below the curves.

In order to understand the structural origin of the high crack resistance and indent shrinkage behavior, the structural details of the glass surface were investigated using micro‐Raman spectroscopy. This technique allows us to obtain local structural information within the indents of borate‐based glasses, which feature several Raman active bands.^43^ However, we note that the Raman spectrum of the pristine glass is convoluted and not all features can be unequivocally assigned to specific structural units (Figure S11 in the Supporting Information). Comparison of the Raman spectra (**Figure** **4**a) recorded on the glass surface indicates that the surface becomes hydrated by exposure to ambient humid air, which manifests itself through an increase in the intensity of the broad envelope at ≈3400 cm^−1^, typically assigned to hydroxyl groups.^44^ On the other hand, there is a concurrent decrease in the intensity of the sharp envelope at ≈780 cm^−1^, which is assigned to various borate superstructures.^43^ Aging the glass surface in a dry desiccator significantly suppresses the extent of surface hydration. This has also been confirmed by differential scanning calorimetry and thermogravimetric analyses (Figure S12 in the Supporting Information). Moreover, the rapid hydration of the glass surface is in agreement with its poor chemical durability, as determined by weight loss measurements, compared to other types of oxide glasses (Figure S5 in the Supporting Information).

Raman spectra acquired in the center of the Vickers indents produced at various loads (Figure 4a) in an aged sample (7 days at room temperature, ≈50% RH) show that the material underneath the point of indenter tip contact is hydrated more quickly compared to the surface, with the extent of hydration increasing with increasing load. This is also shown in Figure 4b, which presents the indentation load dependence of the intensity of the two Raman bands at ≈780 and ≈3400 cm^−1^ (relative to the intensity of the band at 2330 cm^−1^, which is unaffected by indentation or aging). To further support this observation, we show Raman spectra of a freshly polished surface, an aged surface, a 20 N indent placed in the aged surface, and an aged 20 N indent placed in the freshly polished surface (Figure 4c). These spectra show that the glass structure changes by exposure to humid air, as well as due to sharp‐contact loading, with indentation accelerating the surface aging. That is, the area of the ≈3400 cm^−1^ band increases relatively to that of the ≈780 cm^−1^ band with aging time and upon loading, i.e., the indentation loading accelerates the aging‐induced structural changes. We thus conclude that the highly stressed glass network is more vulnerable to the attack of water from the environment. This agrees with previous studies on crack tips in oxide glasses, which are believed to propagate faster through the material once water can hydrolyze the strained network bonds.^33^, ^34^

## 3 Discussion

**Figure 5:**
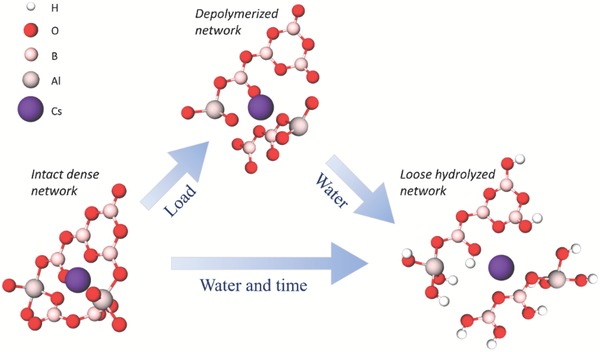
Proposed molecular‐scale deformation mechanism for volume recovery, and in turn ultra‐high crack resistance. The glass network in the pristine glass consists of trigonal B species and tetrahedral Al species with large Cs cations filling out the voids. When a load is applied, movement of atoms is necessary, leading to breakage of network bonds, and as a result, nonbridging O atoms are formed with both Cs and Al cations aiding charge compensation. The deformed structure is more vulnerable to water attack than the nonindented one, hydrolyzing the weakened parts of the network, and the absorption and penetration of hydrous species cause a volume expansion of the network. Eventually, this leads to the observed macroscopic volume recovery of the indent. Such expansion could lead to compressive stresses in the contact area, thus counteracting crack initiation.

In the following, we propose a molecular‐scale deformation mechanism to account for the macroscopic shrinkage of the indentation impressions along with the structural changes of the surface based on the Raman spectroscopy analysis. The pristine glass consists of borate and aluminoborate structural units with large Cs cations encapsulated within the network (**Figure** **5**). When the glass is subjected to stress, the network is disturbed and the borate superstructures break down (see evolution of ≈780 cm^−1^ Raman band in Figure 4b).^14^, ^45^ Consequently, the borate units are depolymerized into smaller ones, with Cs and possibly Al cations providing charge stabilization of the nonbridging O anions. Due to its depolymerized nature, this new structure is more prone to react with water, aiding water molecules to diffuse into the network and hydrolyze the borate units. This is consistent with the differences between the Raman spectra acquired on pristine and indented surfaces of the glass (Figure 4a).

Water molecules penetrating the glassy network are expected to increase the packing density by filling up the empty interstices in the network (similar to traditional alkali modifiers), and at the same time to hydrolyze the oxide network.^46^ These phenomena should have positive and negative effects on hardness, respectively. In the case of the caesium aluminoborate glass, the hardness of the material around the Vickers imprint remains unchanged. On the other hand, the shape of the new indent, placed close to the large aged indent, looks different compared to those more distant from the edge of the aged indent (Figure S10 in the Supporting Information), suggesting some degree of structural change in the deformation zone. When the glassy network is hydrated and is sufficiently flexible to relax around the hydrous species (as in the case of the very soft aluminoborate glass), the network should expand, driven by a structural relaxation toward lower potential energy state (Figure 5), corresponding to relaxation of the indent cavities. Furthermore, the incorporation of water into the glass surface could impose compressive stresses in the indented contact area, as the expanding material cannot be displaced in the surface plane direction into space that is already occupied by the glass itself. This would help to suppress crack formation and growth (similar to the process of ion exchange),^8^ thus, at least partly, accounting for the ultra‐high crack initiation resistance. In addition, water diffusing into the glass network may result in fast stress relaxation.^47^ The caesium aluminoborate network is already intrinsically flexible, yet has high Poisson's ratio compared to other oxide glasses and high polarizability of Cs^+^ ions, which promotes shear‐induced plasticity at room temperature, and more easily dissipates the indentation‐induced work. The presence of hydroxyl groups in the glass structure thus further increases the cracking resistance, as seen from the large difference in crack resistance between as‐polished and aged samples (Section 2.2).

In addition to the role of surface hydration in enabling ultra‐high crack resistance, we note that other alkali and alkaline earth aluminoborate glasses also exhibit relatively high resistance to indentation cracking.^14^, ^25^, ^26^ Their common feature is that the coordination numbers of the network‐forming nuclei (B and Al) are adaptive to external stresses. The energy supplied during a contact event such as indentation can therefore be dissipated by the local speciation change. However, aging of, e.g., a lithium aluminoborate glass^14^ does not lead to the ultra‐high CR value observed herein, because it features higher chemical durability than the present glass. Moreover, phosphate glasses, which are not self‐adaptive but undergo rapid hydration, are generally not damage resistant.^48^ As such, our results suggest that it is the combination of a self‐adaptive network with few nonbridging oxygens and easy surface hydration that gives rise to the micro‐ductile deformation behavior. However, we also note that the low chemical durability of the present glass is disadvantageous for most industrial applications. Such applications might be enabled by coating the glass surface following hydration. Furthermore, it would be interesting in future work to understand if other structural features besides OH‐groups can be used to induce easy shear deformation in the surface layer of oxide glasses.

## 4 Conclusions

Upon surface aging, the caesium aluminoborate glass investigated herein exhibits a significantly higher resistance to indentation cracking (≈400 N) compared to all other known oxide glasses. This results from a combination of a high atomic self‐adaptivity of the glassy network, few nonbridging oxygen atoms, and easy shear‐induced plasticity, resulting from its low chemical durability. The ultra‐high crack resistance observed for this glass demonstrates the hidden potential in using rational composition design instead of existing post‐treatment technologies to improve the damage resistance and micro‐ductilityof glasses. In addition, the shrinkage of the indent cavities in atmospheric air enables their almost full recovery. This behavior, which we have associated with a two‐step mechanism, should also be beneficial for applications, as small surface flaws appear to be able to self‐heal. Overall, we believe that the present discovery paves a new avenue toward development of commercially relevant ductile and self‐healing oxide glasses, which would revolutionize the glass industry.

## 5 Experimental Section

*Sample Preparation*: A bulk glass of 25Cs_2_O–20Al_2_O_3_–55B_2_O_3_ molar composition was synthesized by the melt‐quenching technique. Adequate amounts of Cs_2_CO_3_ (SigmaAldrich, >99.9%), Al_2_O_3_ (SigmaAldrich, >99.5%), and H_3_BO_3_ (Honeywell, >99.5%) powders were weighed and thoroughly mixed. Due to excessive foaming, the mixture was added stepwise to a PtRh crucible and heated to ≈800 °C in an electric furnace (Entech). Once the entire batch was transferred to the crucible, the temperature was raised to 1050 °C. The liquid was allowed to homogenize for 2 h, and then quenched onto a brass plate. The vitrified sample was immediately transferred to an annealing furnace, annealed at 415 °C for 30 min, and slowly cooled down to room temperature. For comparison with the aluminoborate glass, we also included a soda‐lime‐silica float glass (13Na_2_O–6MgO–10CaO–71SiO_2_, in mol%) provided by VELUX A/S and an aluminosilicate glass (12Na_2_O–6MgO–18Al_2_O_3_–6B_2_O_3_–58SiO_2_, in mol%) taken from a previous study.^31^

Appropriate glass samples were cut from the bulk piece for X‐ray diffraction, UV‐vis spectroscopy, simultaneous thermal analysis, Raman spectroscopy, and dissolution rate experiments (see the Supporting Information). The remaining glass was cut into specimens of dimensions suitable for the subsequent mechanical and structural analyses (at least 1 cm^2^ of area and 4 mm of thickness). These were ground and polished using abrasive SiC disks (up to grit 4000) in ethanol. The specimens were then kept in a desiccator to avoid hydration of the surface.

*Mechanical and Structural Characterization*: Density was determined using Archimedes principle of buoyancy by weighing a specimen in air and in ethanol. Longitudinal and transverse sound wave velocities were measured by means of an ultrasonic echography technique using 20 MHz transducers (38DL Plus, Olympus). Based on density and the sound velocities, elastic moduli and Poisson's ratio were determined.

Four series of indentation experiments were performed, entitled I through IV. For Series I, the experiments were performed on a microindenter (CB500, Nanovea) using different diamond tip geometries (sphere, Vickers, and cube corner). The target load (5 N), loading rate (50 N min^−1^), and dwell time (15 s) were determined prior to each indentation cycle, and the resulting indent impressions were recorded. The sample surface of the caesium aluminoborate glass was ground and polished immediately prior to a series of indentation cycles to minimize the effect of hydration, while the soda‐lime‐silica and aluminosilicate glasses were indented without any surface grinding or polishing. The experiments were performed under ambient conditions (room temperature, ≈50% RH, where RH is relative humidity). For Series II, indentations were performed that were intended for subsequent Raman spectroscopy measurements (see below). These were done using the same instrument and conditions as for Series I, but using only the Vickers geometry tip. Moreover, the surface of the caesium aluminoborate glass was either polished immediately before the indentation, or aged for 1, 2, or 7 days under ambient conditions (room temperature, ≈50% RH) prior to indentation. For Series III, indentation experiments were performed on an instrument with higher maximum load (Durascan, Struers), enabling Vickers indentation up to 490 N (50 kgf). These experiments were performed on the caesium aluminoborate glass surface that was kept in ambient conditions for 7 days (room temperature, ≈50% RH), and no surface polishing was conducted immediately prior to indentation. Although these indentation experiments were also performed in ambient conditions, they were done in a different laboratory where the relative humidity was ≈40% RH. For Series IV, the indentation experiments (Durascan, Struers) were done to prepare samples for the laser scanning microscopy analysis (see below). Indents were produced at loads of 0.98, 4.91, and 19.6 N. These indentations were also done at room temperature and ≈40% RH. The caesium aluminoborate surface was polished 24 h prior to the experiment and kept in a desiccator to minimize hydration.

An InVia Renishaw Raman spectroscope was used to acquire Raman spectra ranging from 100 to 4000 cm^−1^ with an excitation time of 10 s using a 532 nm green HeNe laser. The laser spot was focused on the surface of the glass or at the bottom of a Vickers indent (series II indents). Baseline‐subtracted spectra were then normalized with respect to their area.

Laser scanning microscopy (Lext OLS 4100, Olympus) experiments were performed on series IV Vickers indents to acquire topographical images of the indent sites. The exact time between a single indentation experiment and the initiation of the topography scan was recorded. The topographical images were then treated using SPIP software to adjust the tilt of the specimen, extract the cross‐sections of the indents, and compute the indent cavity volume.

## Conflict of Interest

The authors declare no conflict of interest.

## Supporting information

Supplementary
