# Supplementary material for: Breaking the Limit of Micro‐Ductility in Oxide Glasses
Source: Adv Sci (Weinh). 2019 Jul 24;6(18):1901281. doi: 10.1002/advs.201901281 (PMC6755546; doi:10.1002/advs.201901281)
Supplement: Supplementary file 1 — Supplementary [file ADVS-6-1901281-s001.pdf]

Copyright WILEY-VCH Verlag GmbH & Co. KGaA, 69469 Weinheim, Germany, 2019.

ADVANCED SCIENCE
Open Access

Supporting Information

for *Adv. Sci.,* DOI: 10.1002/advs.201901281

Breaking the Limit of Micro-Ductility in Oxide Glasses

*Kacper Januchta, Malwina Stepniewska, Lars R. Jensen, Yang Zhang, Marcel A. J. Somers, Mathieu Bauchy, Yuanzheng Yue, and Morten M. Smedskjaer**

Copyright WILEY-VCH Verlag GmbH & Co. KGaA, 69469 Weinheim, Germany, 2019.

Supporting Information

Breaking the Limit of Micro-Ductility in Oxide Glasses

*Kacper Januchta, Malwina Stepniewska, Lars R. Jensen, Yang Zhang, Marcel A. J. Somers, Mathieu Bauchy, Yuanzheng Yue, Morten M. Smedskjaer**

Supporting Methods

X-ray diffraction. To confirm the amorphous nature of the melt-quenched and aged glass, a fraction of the bulk ceasium aluminoborate sample was cut and crushed into a fine powder using an agate mortar for x-ray diffraction experiments (Empyrean XRD, PANalytical with Cu $K_{\alpha 1}$ radiation of λ = 1.54098 Å). The powder was scanned in spinning mode in the 5° to 80° angle range with a 0.01° step size. The scan was performed thrice on the same sample.

UV-VIS spectroscopy. The optical transparency of the caesium aluminoborate glass was investigated on a 2.2 mm thick specimen, which was polished using SiC paper (up to grit 4000). It was then inserted into a UV-VIS spectrometer (Cary 50 Bio, Varian), and the absorbance at wavelengths between 270 and 800 nm was recorded. Five distinct areas of the specimen were tested to ensure the sample homogeneity. The probed areas were at least 1 mm away from each other.

Differential scanning calorimetry. To determine the calorimetric glass transition temperature (T_g), a small sample was cut from the bulk caesium aluminborate glass piece and grinded to a ~20 mg flat disc for differential scanning calorimetry (DSC). The disc was inserted into a PtRh crucible and heated in the calorimeter (STA 449C, Netzsch) to 850 °C at 10 °C/min in argon. The heat flow curve was recorded to detect the exothermal and endothermal phase transitions. The scan was preceded by a baseline correction (i.e., using an empty crucible), and measurement of for a standard sapphire sample with known isobaric heat capacity. The isobaric heat capacity curve for the glass

was determined by dividing the heat flow curves of the glass sample and the sapphire sample. T_g was then determined as the intercept of the extrapolated isobaric heat capacity of the glass and the tangent at the inflection point of the endothermic glass transition peak.

Raman spectroscopy. The homogeneity of the glass was investigated by acquiring four micro-Raman spectra ranging from 100 to 1600 cm^{-1} (InVia, Renishaw) using a 532 nm green HeNe laser with a 10 sec excitation time. The distance between the four locations used for spectrum acquirement was at least 1 mm. The spectra were subjected to baseline subtraction and normalization with respect to their area.

Chemical durability. To compare the chemical durability (dissolution rate) of the caesium aluminoborate glass with other types of oxide glasses, we cut six rectangular specimens from the bulk glass. Their faces were polished in ethanol using SiC paper (grit up to 4000). The specimens were then ultrasonicated in distilled water for 5 min, and then in acetone for 5 min, followed by drying at 105 °C. The mass and surface area of each specimen were recorded. The specimens were then immersed in aqueous solutions at pH 2, 7, and 14 (pH adjusted by HCl and KOH). The specimens were systematically withdrawn from the aqueous solutions, dried at 105 °C, weighed, and re-immersed in the solutions. The dissolution rate at a given pH was determined from the correlation between mass loss (normalized to the initial surface area) and time.

Synthesis and characterization of lanthanum zinc borate glass. To evaluate whether the present caesium aluminoborate glass features ultra-high crack resistance due to its high Poisson's ratio (0.32), we compare with another oxide glass with similarly high Poisson's ratio. Based on recent studies, showing that certain zinc borate glasses feature relatively high Poisson's ratio[1,2], we here design a lanthanum zinc borate glass, since we have also found that La_2O_3 addition increases the Poisson's ratio[3]. Specifically, we synthesized a $10La_2O_3$ - 45ZnO - $45B_2O_3$ (in mol%) glass by the melt-quenching method. A mixture of La_2O_3 (SigmaAldrich, purity>99.9%), ZnO (VWR, >99.7%),

and H_3BO_3 (Hobeywell, >99.5%) was added to a PtRh crucible and melted at ~1100 °C for 2 hours to form a homogeneous liquid. The hot melt was then quenched onto a brass plate at room temperature. The glass was immediately transferred to a preheated furnace for annealing at its glass transition temperature (560 °C, determined using DSC), and cooled to room temperature at ~3 °C/min rate. The glass piece was then cut to smaller size specimens for Vickers micro-indentation (CB500, Nanovea) and ultrasonic echography (38DL Plus, Olympus) experiments. A ~1 cm^2 specimen of ~2 mm thickness was grinded to be co-planar and polished in ethanol using SiC paper (up to grit 4000). A Vickers micro-indent was produced on the freshly polished surface with a 50 N/min loading and unloading rates, and 15 s dwell time at 5 N. The indent image was captured immediately after unloading, and 3 days later. Several repetitions were made to ensure that the glass consistently exhibited radial cracking. The velocities of the longitudinal and transverse waves were determined from the specimens thickness (measured by a digital micrometer) and the delay time between an impulse (induced by 20 MHz transducers on the surface of the glass) and the echo. Poisson’s ratio was then calculated to be 0.32 from the two sets of sound wave velocities.

Simultaneous thermal analysis. In order to probe the water uptake of the caesium aluminoborate glass, simultanoues thermal analysis (STA 449C, Netzsch) experiments were performed to obtain mass loss and heat flow data. Two small specimens (~500 mg) of the caesium aluminoborate glass were cut from the bulk piece. Each of them was grinded in an agate mortar to a fine powder. One of the specimens was then kept in a desiccator, while the other was kept in an open container stored under ambient conditions (room temperature, ~50% RH). Both specimens were stored for one week. An empty PtRh crucible was heated in the STA instrument to 470 °C at 10 °C/min in argon. Then, ~30 mg portions of the two glass powders were transferred to the crucible, and heated using the same program. The heat flow and mass loss curves were recorded.

Supporting Figures

Figure S1. X-ray diffraction patterns of the caesium aluminoborate glass. Each curve is a sum of three scans in the 5° to 80° range on a powdered sample in spinning mode (Supporting Methods). Both as-prepared (powder made from freshly polished sample) and aged (powder aged for 7 days at room temperature, ~50% RH). For both samples, no sharp Braggs peaks are observed, confirming the amorphous nature of the caesium aluminoborate at least within the XRD detection limit.

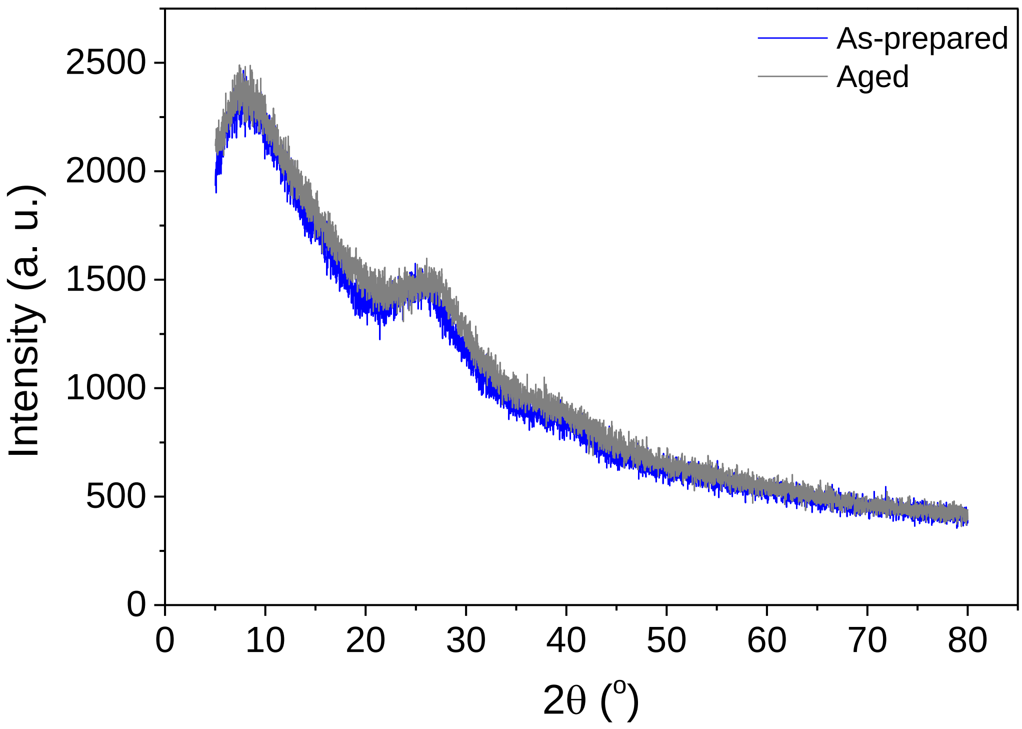

Figure S2. Isobaric heat capacity (C_g) as a function of temperature of the caesium aluminoborate glasses. The differential scanning calorimetry experiment (Supporting Methods) reveals an endothermal glass transition with an onset (i.e., T_g) around 403 °C, followed by what appears to be three exothermal crystallization peaks, and an endothermal melting peak, upon heating.

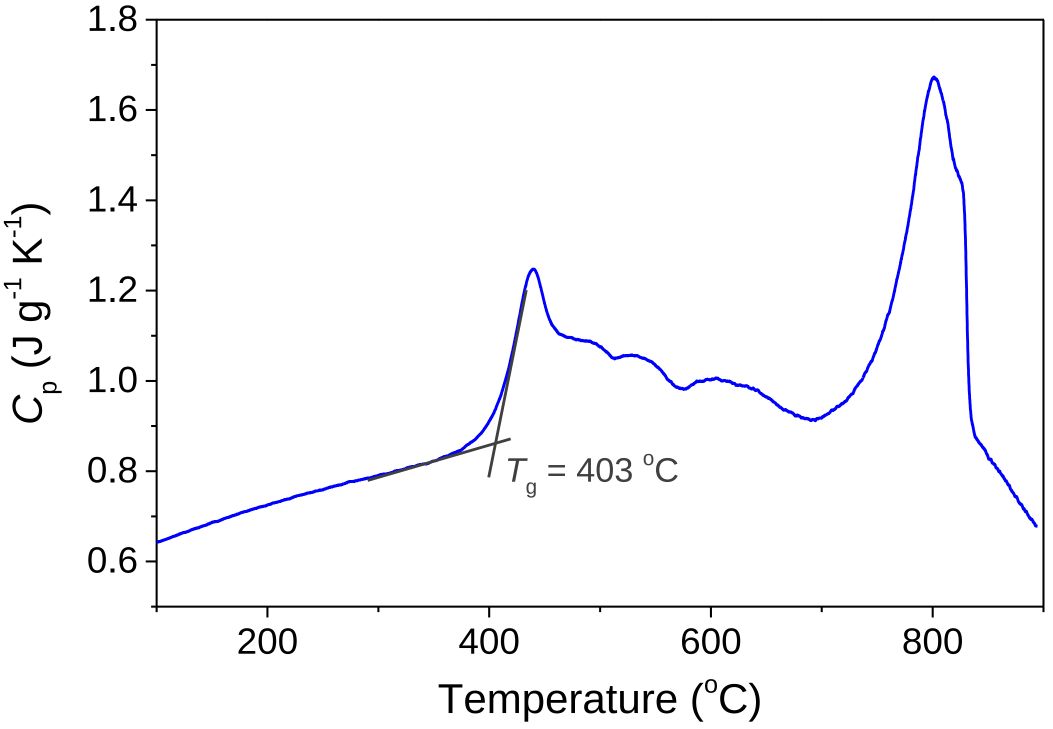

Figure S3. UV-VIS transmittance spectrum of the caesium aluminoborate glass (normalized to 1 mm thickness). The freshly polished glass is transparent in the visible light, with an observed decrease in transmittance upon aging (1 day at room temperature, ~50% RH). Five distinct places on the specimen were probed to ensure homogeneity (Supporting Methods).

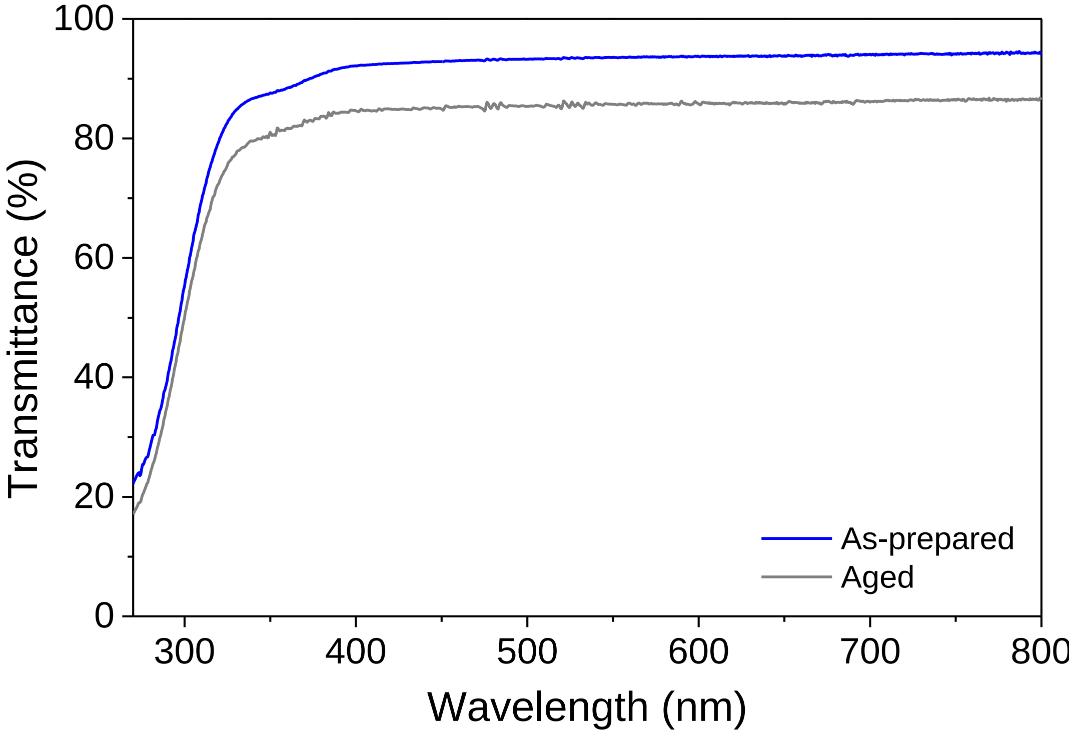

Figure S4. Sample homogeneity as assessed by micro-Raman spectroscopy. No significant differences across the sample surface are observed, suggesting that the glass is compositionally homogeneous (Supporting Methods).

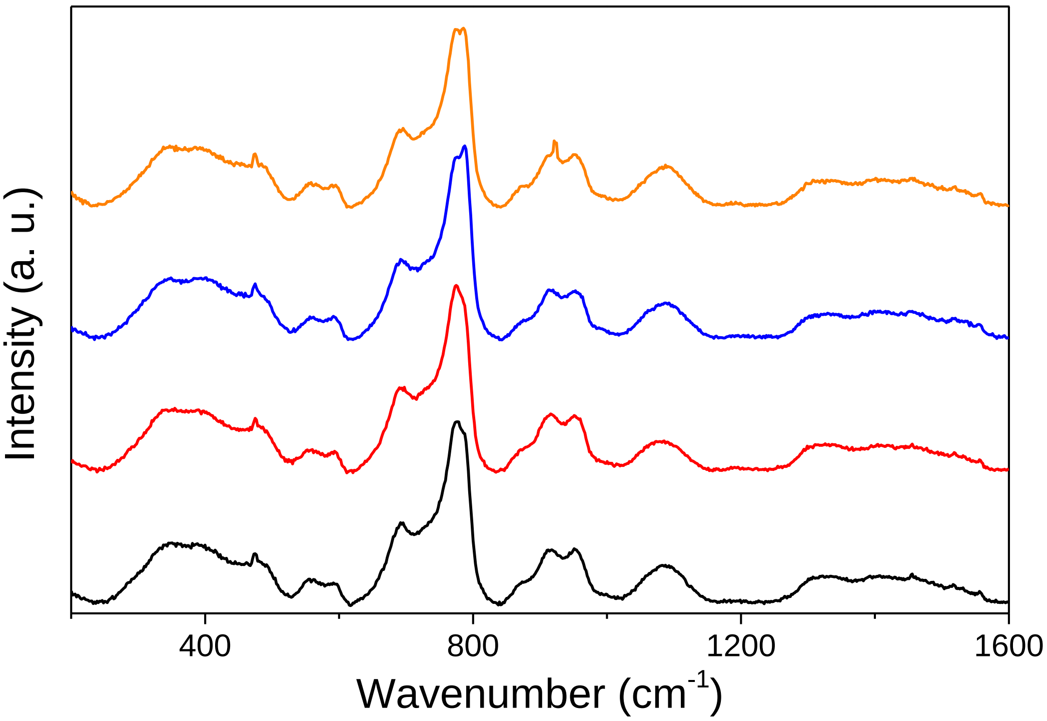

Figure S5. Chemical durability of the caesium aluminoborate glass compared to various oxide glasses. The dissolution rate (D_r) in a neutral pH aqueous solution (Supporting Methods) is much higher for the caesium aluminoborate glass (Cs AlB) compared to other glass networks. The molar compositions of the glasses included here for comparison are (with ID in brackets): $36Na_2O$ – 64 P_2O_5 (P), $30Na_2O$ – $20SiO_2$ – $50P_2O_5$ (SiP), 50CaO – $5B_2O_3$ – $45P_2O_5$ (BP), $15Na_2O$ – 10CaO – $15B_2O_3$ – $75SiO_2$ (BSi), $15Na_2O$ – 10CaO – $15Al_2O_3$ – $75SiO_2$ (AlSi), and $15Na_2O$ – $17Al_2O_3$ – $5B_2O_3$ – $63SiO_2$ (AlBSi). D_r data for these other glasses (i.e., besides Cs AlB) are taken from a previous study, in which D_r was measured under the same conditions.[4] The dissolution rate in acidic and basic conditions follows the same trend, but with different absolute values (not shown).

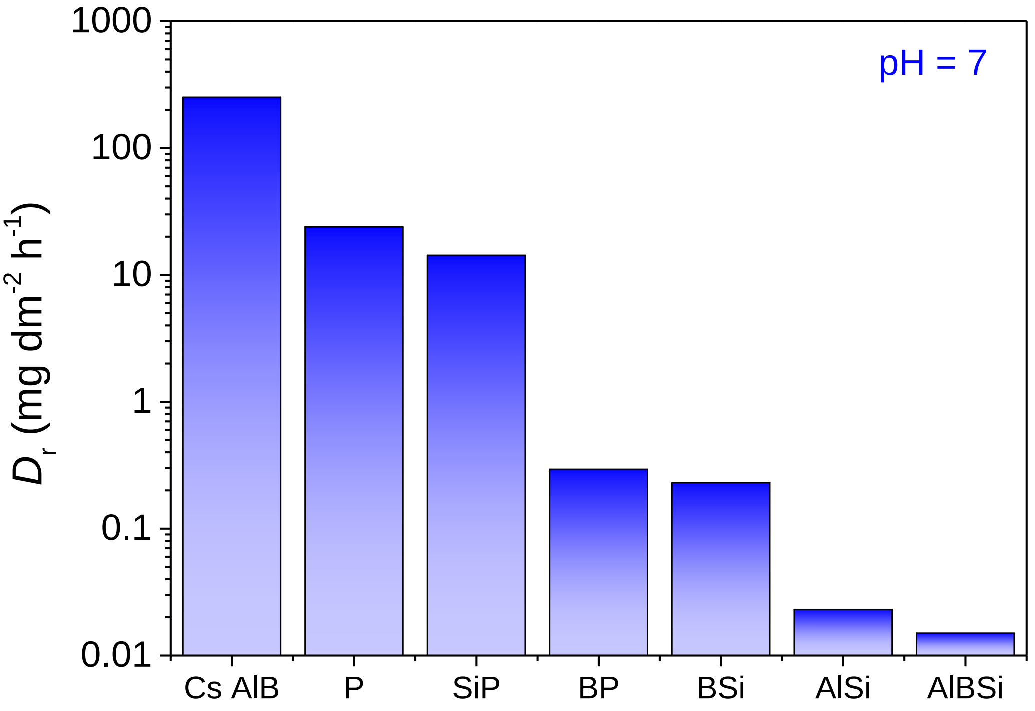

Figure S6. Indentation response of $10La_2O_3$ – 45ZnO – $45B_2O_3$ glass. The Poisson's ratio of the glass was here measured to be 0.32 using ultrasonic echography. a) Vickers indentation at 5 N results in initiation of radial cracks. b) Shape of the indent cavity after being exposed to ambient conditions (room temperature, 50% RH) for 3 days, with no visual changes. The indentation response of the lanthanum zinc borate glass suggests that the high crack resistance and indent cavity shrinkage of the present caesium aluminoborate glass are related to its specific chemical composition and structural details, and not solely to its high Poisson's ratio.

a **b**

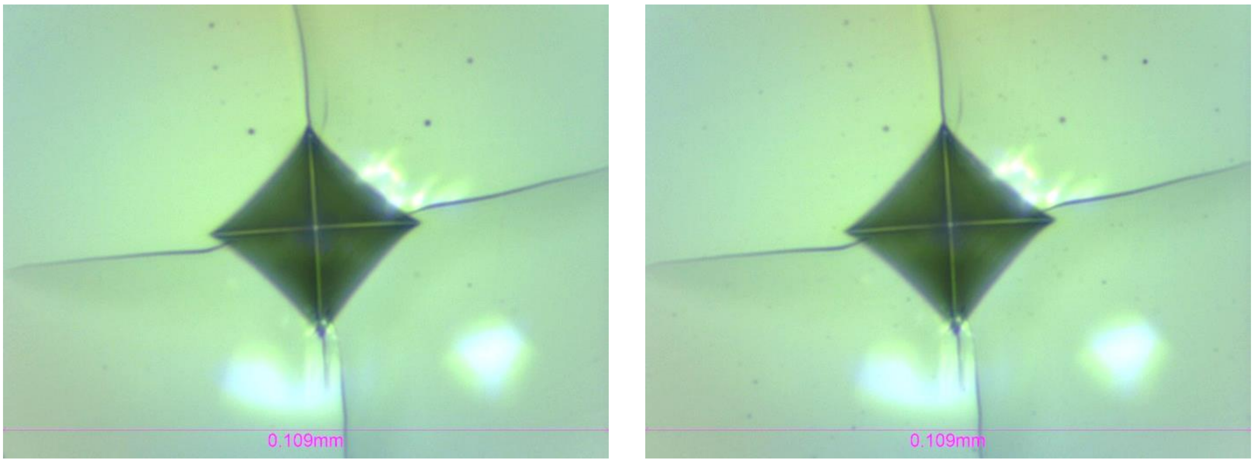

Figure S7. Loading rate dependence of the Vickers indent shape of the caesium aluminoborate glass. a) Vickers indent produced at 20 N peak load with 0.2 N/min loading rate. b) Vickers indent produced at 20 N peak load with 200 N/min loading rate. None of the indents exhibit any radial cracking, but the latter indent exhibits significantly more faults inside the indent cavity. Both indents were produced in a freshly polished surface in ambient conditions (room temperature, ~50% RH), and the images were acquired immediately after unloading.

a **b**

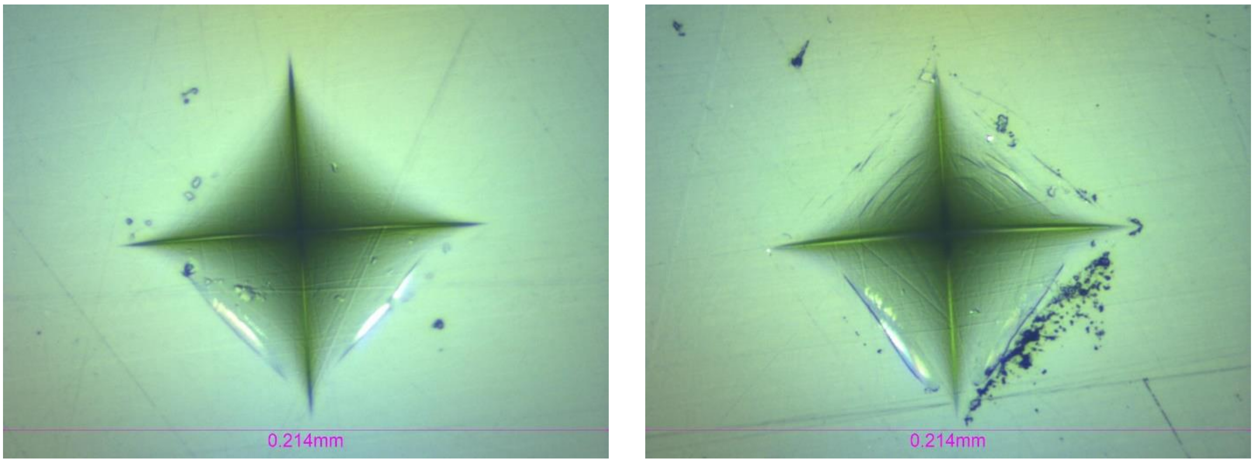

Figure S8. Optical impressions of a 5 N Vickers indent in the caesium aluminoborate glass. Images were acquired a) immediately after unloading, b) after aging in ambient conditions (room temperature, 40% RH) for 1 hour, c) after aging in ambient conditions for 3 days, and d) after aging for 4 months.

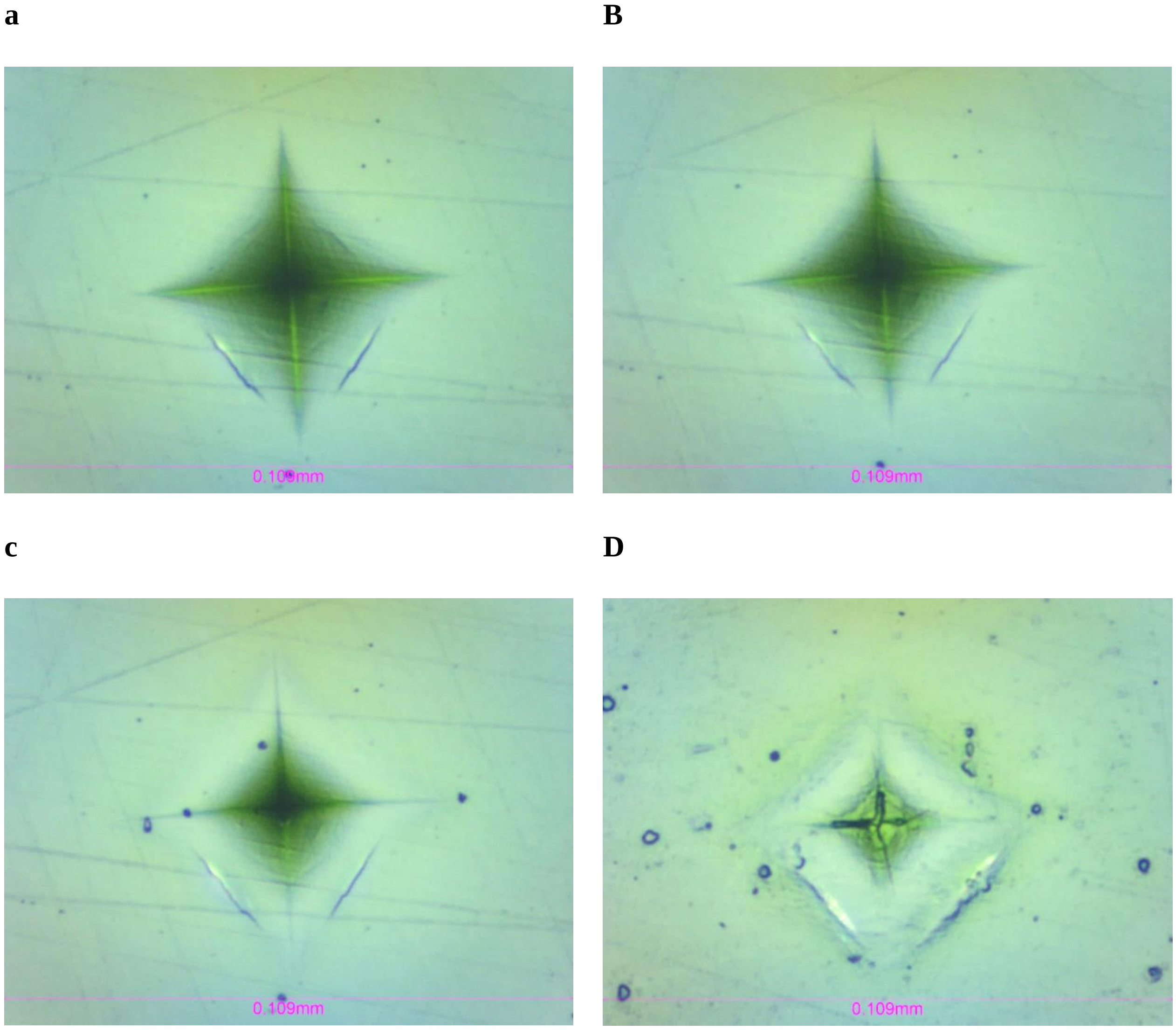

Figure S9. Volume recovery behavior assessed by laser microscopy. 2D-representations of topographical impressions acquired using laser microscopy for 19.6 N Vickers indents aged in ambient conditions (room temperature, ~50 % RH) for a) 21 minutes, b) 46 minutes, c) 99 minutes, d) 230 minutes, and e) 9 days. The images were subjected to a levelling procedure to account for the tilt of the sample employing SPIP™ software. The relative recovery values of the depth and the cavity volume are 2 % and 9 %, respectively, within the 230 minute time interval. The build-up of material around the indent aged for 9 days inhibits accurate evaluation of the depth and volume recovery. The images are 258 μm in horizontal and vertical directions, while the depth is indexed by a color scale next to the images.

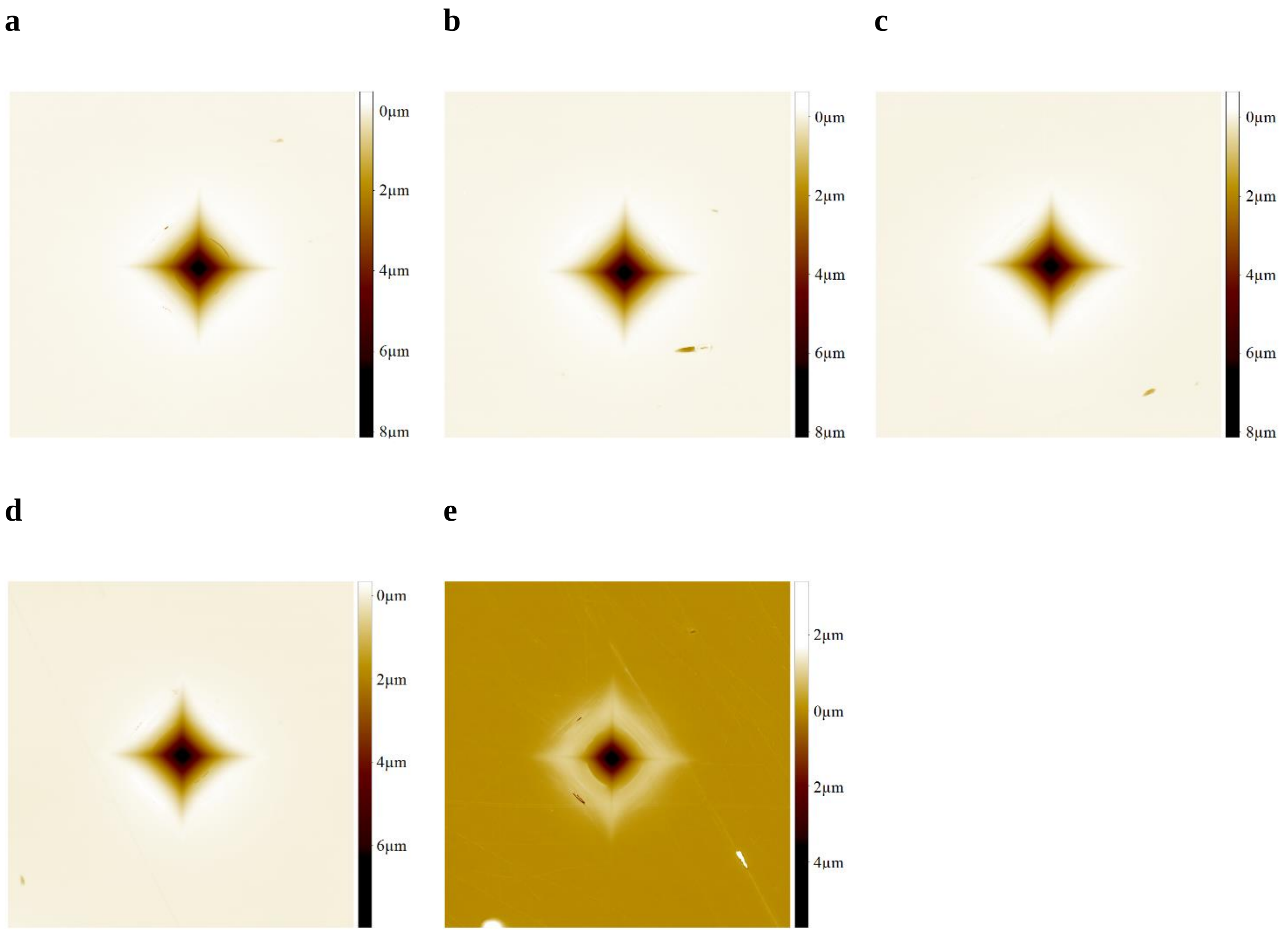

Figure S10. Optical microscopy images of relatively small Vickers indents placed in vicinity of an aged larger indent. a) Overview of the three small 0.5 N indents positioned next to the large 100 N indent. b-d) 0.5 N indents situated at b) 84 µm, c) 149 µm, and d) 213 µm away from the center of the large indent, which was aged in ambient conditions (room temperature, ~40% RH) for 2 months. The images of the 0.5 N indents were acquired immediately after unloading. The 0.5 N indent closest to the center of the aged indent (within the rim of the 100 N indent) appears different than the indents situated further away. More specifically, the bow-in of the sides as well as the bright area surrounding the indent are smaller, although the hardness value is the same within the error of measurement.

a

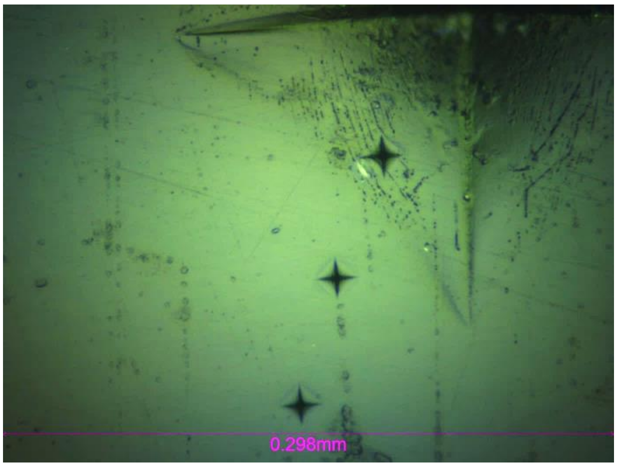

b

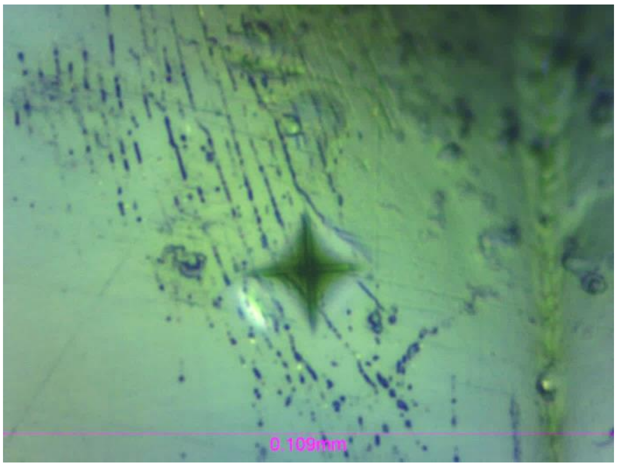

c

d

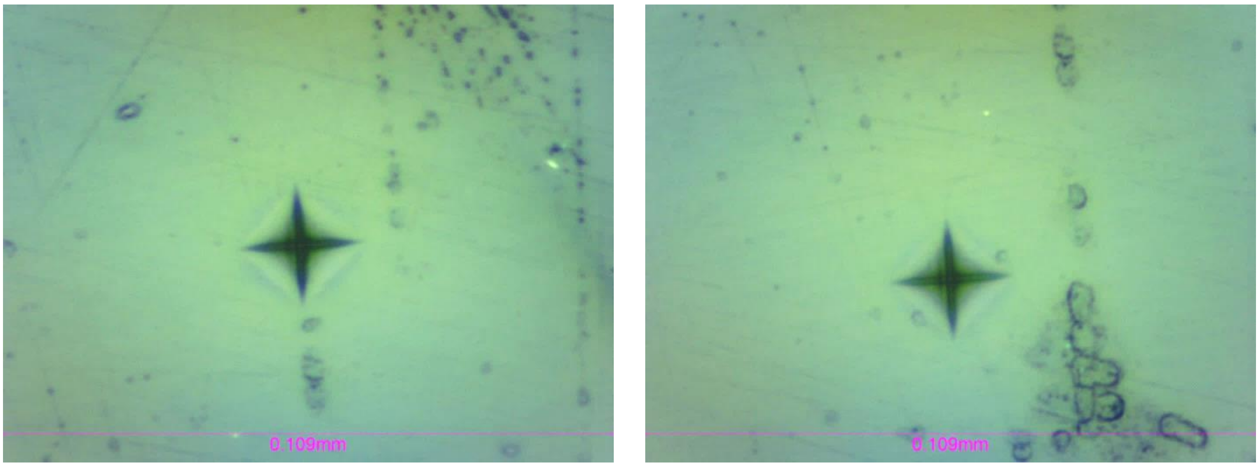

Figure S11. Raman band assignments in the caesium aluminoborate glass. The glass has here been aged in ambient conditions (room temperature, ~40% RH) for one week. The spectrum consists of several broad envelopes.

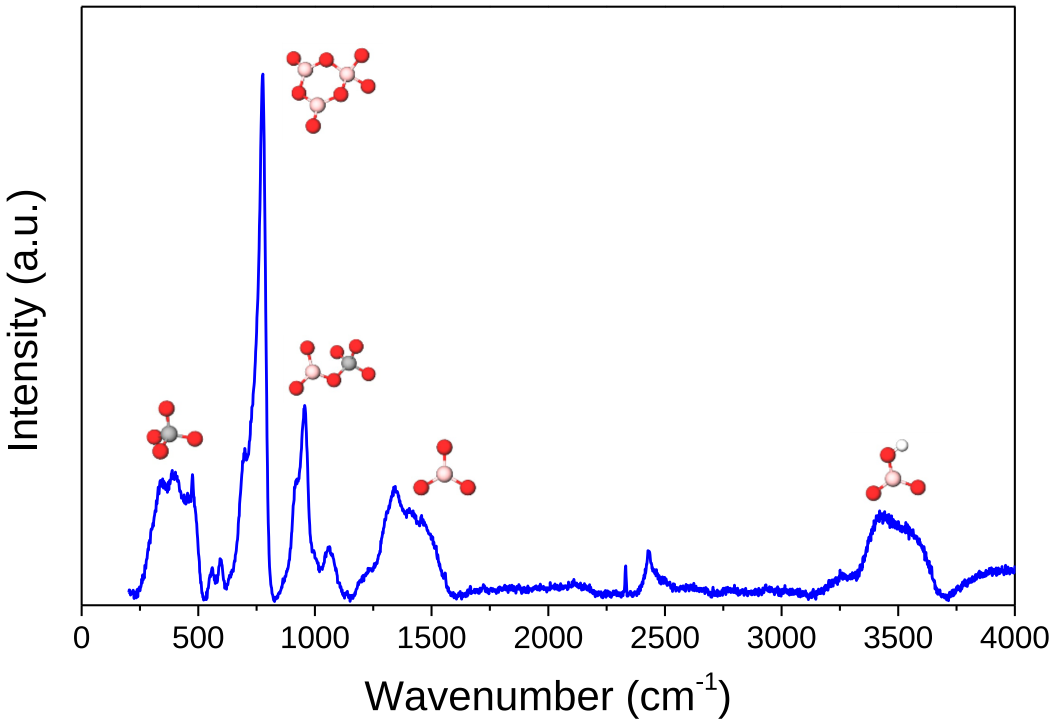

The first band, ranging from ~250 to ~500 cm^{-1}, contains signal contributions from cation-oxygen stretching in tetrahedral Al and B units.[5,6] The second band, ranging from ~600 to ~800 cm^{-1}, corresponds to various superstructural units of the borate network such as, e.g., metaborates, triborates or pentaborates,[7,8] and possibly similar structures involving Al cations. The next band, ranging from ~800 to ~1200 cm^{-1}, is usually assigned to other borate superstructural units such as diborates as well as the more depolymerized pyroborates and orthoborates.[7,8] Signal contributions from a mixed aluminoborate network has also been suggested.[6] Next, the broad band, stretching from ~1200 to ~1600 cm^{-1}, is related to B–O stretching in trigonal BO_3 units.[7] The assignment of the two small bands situated around 2300 and ~2400 cm^{-1} is uncertain, but are probably not related to the glass structure. The former could be due to N_2 in air. Finally, the broad envelope situated between ~3200 and ~3700 cm^{-1} should be associated with hydroxyl groups attached to the glass network resulting from a surface hydration.[9,10]

Figure S12. Water release upon heating as assessed by simultaneous thermal analysis. Both the endothermic peak corresponding to vaporization of water from the heat flow (solid line) and the mass loss (dashed line) suggest that the glass powder aged in ambient conditions (room temperature, ~40% RH) for one week releases much more water compared to the glass powder stored in a desiccator for the same time period. Furthermore, the glass transition peak appears to shift to higher temperatures with increasing water content, suggesting that structural changes occur during hydration. The heat flow and mass loss curves of the bulk caesium aluminoborate glass have been included for comparison as well. Although glass transition temperature is expected to decrease with increasing water content in B_2O_3-rich glasses,[11] the fraction of four-fold coordinated B atoms can in fact increase upon water incorporation.[12] Such structural evolution would impose an increase in the network rigidity, which could cause an increase in the glass transition temperature.

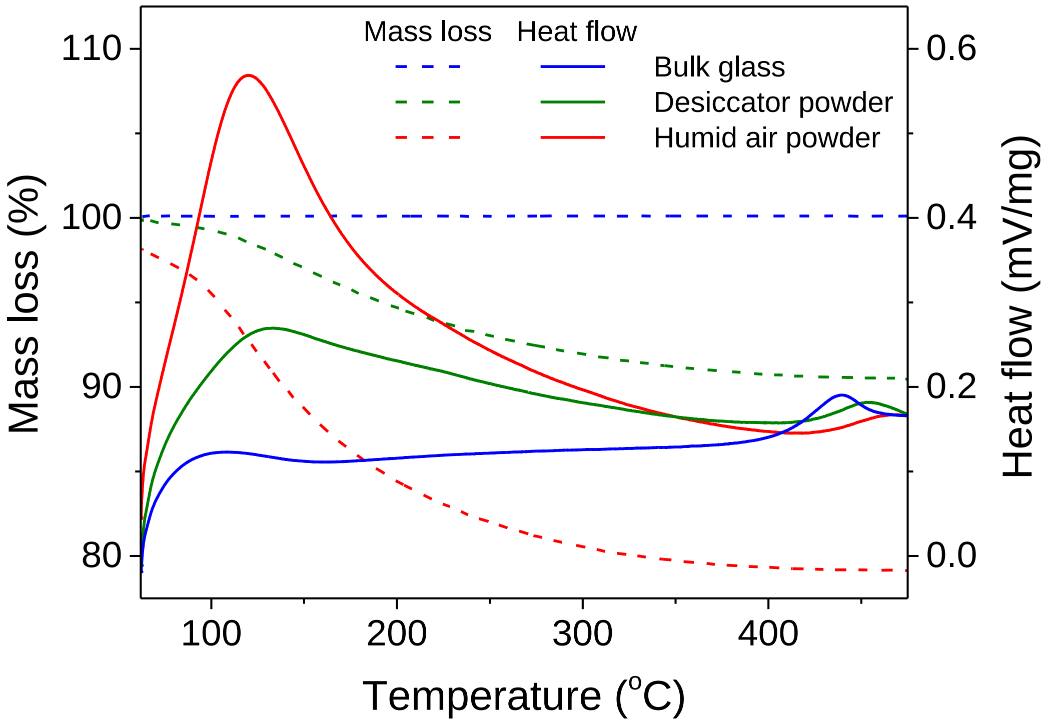

Supporting references

In addition to the literature cited in the main paper, several supporting references are included above. Additionally, we here include the wide range of crack resistance and Vickers hardness data used in Figure 2b from the main paper: borosilicates,[13–17] aluminosilicates,[18–25] silicates,[26–28] aluminoborates,[29–31] and other various oxide glass compositions.[1,3,32–34]

[1] Z. Y. Yao, D. Möncke, E. I. Kamitsos, P. Houizot, F. Célarié, T. Rouxel, L. Wondraczek, *J. Non. Cryst. Solids* **2016**, *435*, 55.

[2] D. Möncke, E. I. Kamitsos, D. Palles, R. Limbach, A. Winterstein-Beckmann, T. Honma, Z. Yao, T. Rouxel, L. Wondraczek, *J. Chem. Phys.* **2016**, *145*, 124501.

[3] K. Januchta, R. Sun, L. Huang, M. Bockowski, S. J. Rzoska, L. R. Jensen, M. M. Smedskjaer, *J. Non. Cryst. Solids* **2018**, *494*, 86.

[4] N. Mascaraque, M. Bauchy, M. M. Smedskjaer, *J. Phys. Chem. B* **2017**, *121*, 1139.

[5] P. McMillan, B. Piriou, *J. Non. Cryst. Solids* **1983**, *55*, 221.

[6] R. K. Brow, D. R. Tallant, G. L. Turner, *J. Am. Ceram. Soc.* **1997**, *80*, 1239.

[7] W. L. Konijnendijk, J. M. Stevels, *J. Non. Cryst. Solids* **1975**, *18*, 307.

[8] E. I. Kamitsos, G. D. Chryssikos, *J. Mol. Struct.* **1991**, *247*, 1.

[9] A. Anedda, C. M. Carbonaro, F. Clemente, R. Corpino, P. Carlo, Ricci, *J. Phys. Chem. B* **2003**, *107*, 13661.

[10] M. Mercier, A. Di Muro, D. Giordano, N. Métrich, P. Lesne, M. Pichavant, B. Scaillet, R. Clocchiatti, G. Montagnac, *Geochim. Cosmochim. Acta* **2009**, *73*, 197.

[11] S. Reinsch, C. Roessler, U. Bauer, R. Müller, J. Deubener, H. Behrens, *J. Non. Cryst. Solids*

2016, *432*, 208.

[12] U. Bauer, H. Behrens, M. Fechtelkord, S. Reinsch, J. Deubener, *J. Non. Cryst. Solids* **2015**, *423–424*, 58.

[13] Y. Kato, H. Yamazaki, Y. Kubo, S. Yoshida, J. Matsuoka, T. Akai, *J. Ceram. Soc. Japan* **2010**, *118*, 792.

[14] S. Yoshida, Y. Nishikubo, A. Konno, T. Sugawara, Y. Miura, J. Matsuoka, *Int. J. Appl. Glas. Sci.* **2012**, *3*, 3.

[15] M. N. Svenson, T. K. Bechgaard, S. D. Fuglsang, R. H. Pedersen, A. O. Tjell, M. B. Østergaard, R. E. Youngman, J. C. Mauro, S. J. Rzoska, M. Bockowski, M. M. Smedskjaer, *Phys. Rev. Appl.* **2014**, *2*, 24006.

[16] R. Limbach, A. Winterstein-Beckmann, J. Dellith, D. Möncke, L. Wondraczek, *J. Non. Cryst. Solids* **2015**, *417–418*, 15.

[17] M. Barlet, J. M. Delaye, T. Charpentier, M. Gennisson, D. Bonamy, T. Rouxel, C. L. Rountree, *J. Non. Cryst. Solids* **2015**, *417–418*, 1.

[18] J. Kjeldsen, M. M. Smedskjaer, J. C. Mauro, Y. Yue, *Appl. Phys. Lett.* **2014**, *104*, 51913.

[19] K. G. Aakermann, K. Januchta, J. A. L. Pedersen, M. N. Svenson, S. J. Rzoska, M. Bockowski, J. C. Mauro, M. Guerette, L. Huang, M. M. Smedskjaer, *J. Non. Cryst. Solids* **2015**, *426*, 175.

[20] M. M. Sebdani, J. C. Mauro, L. R. Jensen, M. M. Smedskjaer, *J. Non. Cryst. Solids* **2015**, *427*, 160.

[21] T. K. Bechgaard, A. Goel, R. E. Youngman, J. C. Mauro, S. J. Rzoska, M. Bockowski, L. R.

Jensen, M. M. Smedskjaer, *J. Non. Cryst. Solids* **2016**, *441*, 49.

[22] H. Morozumi, S. Yoshida, J. Matsuoka, *J. Non. Cryst. Solids* **2016**, *444*, 31.

[23] G. A. Rosales-Sosa, A. Masuno, Y. Higo, H. Inoue, *Sci. Rep.* **2016**, *6*, 23620.

[24] T. M. Gross, J. J. Price, *Front. Mater.* **2017**, *4*, 4.

[25] T. M. Gross, J. Wu, D. E. Baker, J. J. Price, R. Yongsunthon, *J. Non. Cryst. Solids* **2018**, *494*, 13.

[26] C. Hermansen, J. Matsuoka, S. Yoshida, H. Yamazaki, Y. Kato, Y. Z. Yue, *J. Non. Cryst. Solids* **2013**, *364*, 40.

[27] G. Scannell, D. Laille, F. Célarié, L. Huang, T. Rouxel, *Front. Mater.* **2017**, *4*, 6.

[28] P. Sundberg, L. G. Bäck, R. Orman, J. Booth, S. Karlsson, *Thin Solid Films* **2019**, *669*, 487.

[29] K. Januchta, R. E. Youngman, A. Goel, M. Bauchy, S. J. Rzoska, M. Bockowski, M. M. Smedskjaer, *J. Non. Cryst. Solids* **2017**, *460*, 54.

[30] K. Januchta, R. E. Youngman, A. Goel, M. Bauchy, S. L. Logunov, S. J. Rzoska, M. Bockowski, L. R. Jensen, M. M. Smedskjaer, *Chem. Mater.* **2017**, *29*, 5865.

[31] K. F. Frederiksen, K. Januchta, N. Mascaraque, R. E. Youngman, M. Bauchy, S. J. Rzoska, M. Bockowski, M. M. Smedskjaer, *J. Phys. Chem. B* **2018**, *122*, 6287.

[32] S. Striepe, N. Da, J. Deubener, L. Wondraczek, *J. Non. Cryst. Solids* **2012**, *358*, 1032.

[33] P. Sellappan, T. Rouxel, F. Celarie, E. Becker, P. Houizot, R. Conradt, *Acta Mater.* **2013**, *61*, 5949.

[34] S. Kapoor, K. Januchta, R. E. Youngman, X. Guo, J. C. Mauro, M. Bauchy, S. J. Rzoska, M. Bockowski, L. R. Jensen, M. M. Smedskjaer, *Phys. Rev. Mater.* **2018**, *2*, 63603.
